# Ultrafast intersystem crossing dynamics in uracil unravelled by *ab initio* molecular dynamics†
†Electronic supplementary information (ESI) available: Discussion of fitting procedure for decay time constants, assignment of state character for ISC transitions and Cartesian coordinates of molecular geometries. See DOI: 10.1039/c4cp04158e



**DOI:** 10.1039/c4cp04158e

**Published:** 2014-10-10

**Authors:** Martin Richter, Sebastian Mai, Philipp Marquetand, Leticia González

**Affiliations:** a Institute of Theoretical Chemistry , Währinger Str. 17 , 1090 Vienna , Austria . Email: philipp.marquetand@univie.ac.at

## Abstract

*Ab initio* molecular dynamics simulations have been performed in order to investigate the relaxation dynamics of uracil after UV excitation in gas phase. Intersystem crossing (ISC) has been included for the first time into time-dependent simulations of uracil, allowing the system to relax in the singlet as well as in the triplet states. The results show a qualitatively different picture than similar simulations that include singlet states only. The inclusion of ISC effectively quenches the relaxation to the singlet ground state and instead privileges transitions from the low-lying nπ* state (S_1_) to a ππ* triplet state (T_2_) followed by rapid internal conversion to the lowest triplet state.

## Abstract 2

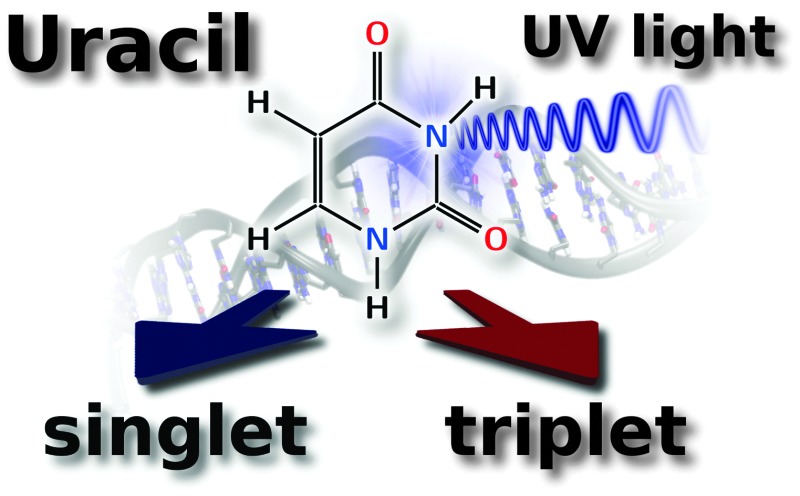
Surface hopping simulations of the RNA nucleobase uracil show that intersystem crossing and hence triplet states play an important role during the photorelaxation after excitation with UV light.

## 1 Introduction

The interaction of ultraviolet (UV) light with deoxyribonucleic and ribonucleic acid (DNA/RNA) can lead to molecular photodamage and ultimately to mutations in the genetic code.^1^ Despite this possibility, DNA and RNA, as well as their building blocks – the nucleobases – are remarkably photostable. This means that, after light irradiation the excited molecules activate photophysical mechanisms that efficiently return the system to the electronic ground state before detrimental excited-state reactions can take place. The quest to understand how these molecules transform the absorbed energy into heat, redistributing it as kinetic energy among all the degrees of freedom and dissipating it into the environment avoiding damage, has become a hot topic.^2–6^ Accordingly, the last decade has witnessed a large number of experimental and theoretical studies on this subject.

Ultrafast time-resolved femtosecond (fs) spectroscopic studies^7–22^ have shown that the relaxation of nucleobases is not a single molecular process but rather a complex one, consisting of several subprocesses, which take place on different time scales. Although the specifics depend on the experimental setup, usually one subprocess is found to correspond to a time constant on the order of fs accompanied by another one on the time scale of a few picoseconds (ps); in some cases, longer subprocesses on the order of nanoseconds (ns) can also be detected.

With the help of extended theoretical methods, it seems now well-established that the observed ultrafast time scales responsible for photostability are the result of decay due to internal conversion (IC) *via* conical intersections (CoIn), which are able to bring the excited molecule from the manifold of electronically excited singlet states to the ground state in a ps or sub-ps time scale.^23–26^ Less clear, however, is the role played by intersystem crossing (ISC) in the photodeactivation of nucleobases.

Static quantum chemical computations have proposed that ISC from the singlet to the triplet manifold should be possible in some of the nucleobases.^27–33^ The direct observation of triplet states is experimentally difficult if their quantum yield is small.^7,34,35^ Moreover, the standard pump–probe setups employed^7,19–21^ do not use a probe wavelength that can detect low-lying triplet states after excitation;^36^ therefore, the presence of triplet states is in most cases inferred indirectly. This has not prevented, however, to correlate triplet states^15,16^ to the long-lived transients observed experimentally. Despite the fact that spin–orbit couplings (SOC) are small in organic molecules and thus ISC is traditionally conceived as a slow process in comparison to IC,^37^ recently our group has demonstrated that, in cytosine, ISC can take place on an ultrafast time scale of few hundreds of fs, hence also contributing to the fs and ps time constants detected experimentally.^26,36,38^ While ISC is astonishingly fast in this case, cytosine seems to be not the only organic molecule where ISC and IC processes can compete on the same time scale. Experimental fast to ultrafast times scales for ISC have been reported for aldehydes,^39^ a number of small aromatic compounds, such as benzene,^40,41^ naphthalene, anthracene and their carbonylic derivatives^42–55^ as well as nitrocompounds.^46,56–68^ The substitution of oxygen by sulfur in nucleobases enhances ISC so dramatically that the ultrafast IC responsible for photostability disappears while turning ISC to the lowest triplet states into the most efficient deactivation pathway – with enormous consequences for photodamage.^64–66,69–74^ The dimerization of pyrimidine nucleobases, one of the most abundant lesions in UV-irradiated DNA, is also claimed to be mediated by triplet states.^35,75,76^

ISC is clearly an important photophysical process; however, dynamical simulations accounting for spin transitions are very much underrepresented in comparison to studies dealing with IC. Spin-induced transitions have been simulated by wave packet propagations along one or few dimensions by Daniel and coworkers in organometallic compounds.^77–87^ Several approaches, from quantum dynamics in reduced dimensions to semiclassical dynamics, have been combined to model and control the ultrafast spin–flip in dihalogens in argon matrices.^88–91^ Surface-hopping methods have been also employed to study ISC in the S + H_2_ reaction.^92^ However, the modelling of dynamical processes including both IC and ISC on the same footing is much more recent, especially in full dimensions. The dynamics of coupled singlet and triplet states have been simulated with a reduced vibronic Hamiltonian for benzene,^41^ HF^93^ and with exact three-dimensional wave packets^94^ as well as with *ab initio* molecular dynamics^95^ for SO_2_. Further semiclassical surface-hopping approaches using semiempirical Hamiltonians have been employed for acetone,^96^ pentanal^55^ and 6-thioguanine,^70^ using time-dependent density functional theory for a few transition metal complexes^97,98^ and using on-the-fly *ab initio* multiconfigurational calculations for the DNA nucleobase cytosine.^36,38^

In this work, we focus on uracil, an RNA nucleobase. Time-resolved experiments in uracil have been first reported in ref. 16 and later on by others.^12,13,15^ Depending on the resolution, one, two, or three time constants have been resolved. The most recent experiments of Kotur *et al.* ^12^ and Matsika *et al.* ^13^ combine time-of-flight mass spectroscopy (TOF-MS) with strong field dissociative ionization, thereby yielding a picture of multiple bifurcations in the deactivation mechanism of uracil. There have been a number of dynamical simulations published dealing with uracil,^24,99–105^ but none includes triplet states. Hence, the present study is designed to fill this gap, showing for the first time dynamics simulations including simultaneously non-adiabatic couplings (which mediate IC *via* CoIn) and SOC (which allows for ISC). We demonstrate that ISC competes with IC and should be taken into account to explain the ultrafast deactivation of uracil after UV irradiation. Further, the simulations presented in this paper aim at completing the knowledge on the fundamental question of which factors on the atomistic level contribute to the photostability of DNA/RNA, ultimately motivating experiments that can time-resolve non-adiabatic dynamics involving triplet states using emerging ultrafast photon technologies.

## 2 Methods

The molecular dynamics simulations on uracil have been carried out using the semiclassical *ab initio* molecular code SHARC,^106^ which is a surface-hopping algorithm^107^ able to deal with arbitrary couplings. Previous dynamical applications of SHARC can be found in ref. 36, 38, 95 and 108–111. This surface-hopping algorithm uses a fully diagonal, spin-mixed electronic basis, resulting from the diagonalization of the Hamiltonian containing non-adiabatic and spin–orbit couplings.^36,106^ The integration of the nuclear motion is done with the velocity-Verlet algorithm^112,113^ with a time step of 0.5 fs for 1 ps; the time evolution of the quantum amplitudes is followed with a time step of 0.02 fs. Decoherence correction was taken into account using the energy-based method of Granucci and Persico with a parameter of *α* = 0.1 hartree.^114^

**Fig. 1:**
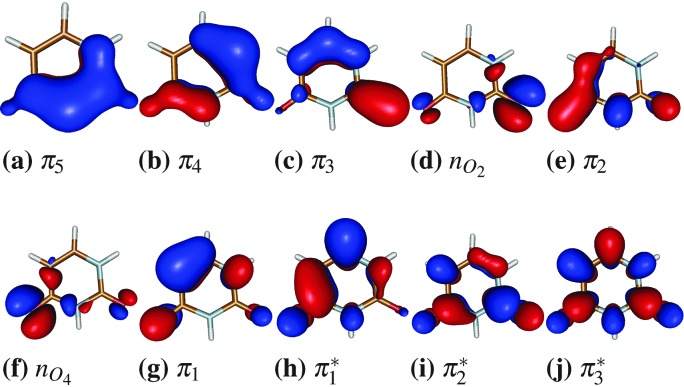
Active space of uracil including 14 electrons in 10 orbitals. The (12,9) active space misses the n_O_2__ orbital.

The electronic energies, gradients, non-adiabatic couplings and SOCs were evaluated on-the-fly for each nuclear integration time step using the complete active space self-consistent field (CASSCF)^115,116^ method and the 6-31G* basis set. Two different active spaces were employed: a (12,9) consisting of 12 electrons in 9 orbitals and a (14,10), with two electrons more in an additional orbital. The latter active space consist of 8 π/π* orbitals as well as 2 n orbitals located at the oxygen atoms of uracil (see Fig. 1), and it has been employed before for quantum chemical calculations^117^ and molecular dynamics in the singlet manifold only.^104,105^ The smaller active space contains one oxygen lone pair less. The excited state properties are calculated using the state-average CASSCF version, including the lowest four singlet states and the lowest three triplet states, *i.e.*, 7 electronic states. Note that in the dynamics the triplet components were treated explicitly, giving 13 states in total. The assessment of the CASSCF energies has been done using single point calculations at the more accurate CASPT2^118,119^ level of theory. All quantum mechanical calculations were performed using the MOLPRO 2012 package of programs.^120^

For the generation of the initial conditions, the ground state equilibrium geometry was optimized at the CASSCF(14,10) or CASSCF(12,9) level of theory, as specified below. Using the corresponding harmonic frequencies, a Wigner distribution of 2000 uncorrelated velocities and geometries has been generated. For each initial condition a single point calculation has been performed to simulate an absorption spectrum from the oscillator strengths, as explained in ref. 121. The oscillator strengths also serve for selecting the number of trajectories to be propagated from each electronic excited state, assuming an instantaneous excitation (a *δ*-pulse).

The evaluation of trajectories is made only on those finishing the whole simulation time of 1 ps, unless specified otherwise, or residing in the S_0_ or T_1_ for at least 15 fs. For the statistical evaluation, we calculated so-called “spectroscopic” populations in addition to the ones in the molecular Coulomb Hamiltonian (MCH)^122^ electronic basis. The transformation from the MCH basis to the spectroscopic one is done in an approximate manner using the transition dipole moment between the electronic ground and the calculated excited states. If the transition dipole moment is >0.05 a.u., the state is considered a bright ππ* state and for values <0.05 a.u. the state is a dark nπ* state. If the transition dipole moment is very small (<1 × 10^–6^ a.u.) the state is assigned to a triplet state. The ground state S_0_ corresponds always to the closed-shell state in the present case and therefore, we use the label S_0_ also in the spectroscopic representation. Statistical analysis of the trajectory data within the spectroscopic representation provides a better description of the experimentally observed decay rates, since the physical properties of the spectroscopic states change less than in the MCH representation.^36^

## 3 Results and discussion

### 3.1 Excitation energies and absorption spectrum

**Fig. 2:**
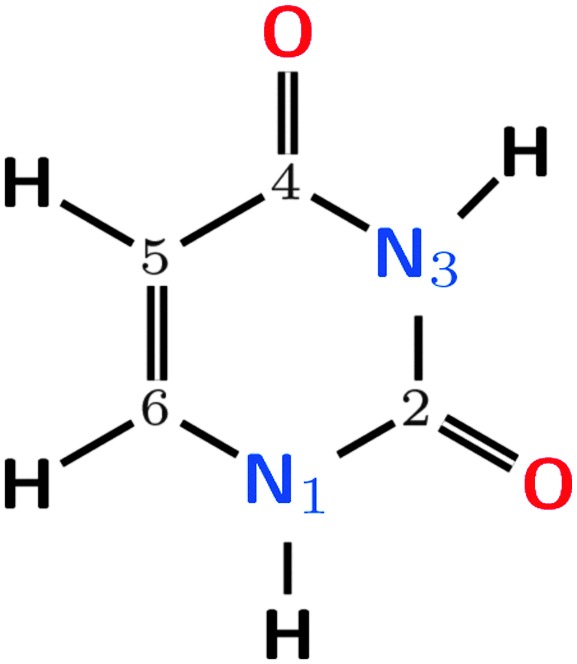
Uracil in its diketo form, with ring atom numbering.

The pyrimidine nucleobase uracil presents 13 different tautomers. Among them, this study focuses on the diketo form (Fig. 2) since it is the biologically relevant and the dominant tautomer in gas phase and solution.^123^

The experimental absorption spectrum of uracil shows its maximum at 244 nm (5.08 eV).^124^ A number of excited state calculations for uracil are reported in the literature. Good surveys can be found in ref. 23 and 125–127. In Table 1, we compile excitation energies obtained at the CASSCF and CASPT2 levels of theory. Singlet and triplet states are given, as available. For all CASPT2 calculations, the band at *ca.* 5.1 eV corresponds to the S_2_ state, which is of ππ* character and possesses the largest oscillator strength. The lowest-lying singlet state is of nπ* character and its energy fits reasonably well within the energetic range recorded experimentally.^128^ Taking as a reference the calculation of Climent *et al.*,^29^ who use CASPT2/CASSCF(14,10) and averaging separately singlet (SA4) and triplet (SA3) states, one can see that the nature of the S_3_ state is more sensible to the level of theory. Using this SA4/SA3-CASPT2/CASSCF(14,10) protocol, the first excited dark nπ* state is followed by two ππ* states, whereas a SA7-calculation (where singlet and triplets states are averaged together) predicts a second nπ* state as the S_3_. The removal of one n-orbital (the n_O_2__) from the active space (resulting in an (12,9) active space) yields the same order of states as in Climent *et al.* ^29^ The use of symmetry to obtain the A′ and A′′ states separately, as done in the benchmark paper of Schreiber *et al.*,^125^ has a minor effect in the CASPT2 energies. The inclusion of dynamical correlation when going from CASSCF to CASPT2, in contrast, is more important, since it affects the singlet ππ* states more dramatically than the nπ* ones, thereby influencing greatly the energetic position of the singlet states in uracil. While S_1_ and S_2_ are almost degenerated at CASPT2, the destabilization of the ππ* at CASSCF separates the states by more than 1 eV, making in turn the S_2_ and S_3_ very close in energy. The triplet states seem to be more robust with respect to the level of theory, both regarding the active space and the inclusion of dynamical correlation. The T_1_ is a ππ* state located at around 4 eV, well-separated energetically from an nπ* and a second ππ* state.

**Table 1:** Vertical excitation energies (in eV) of uracil at CASSCF and CASPT2 level of theory with different active spaces. State characters are also indicated and oscillator strengths are given in parentheses

State	Experiments	CASPT2				CASSCF		
		SA4/SA3	SA7	SA7	SA5/SA4	SA4/SA3	SA7	SA7
		(14,10)	(14,10)	(12,9)	(10,8)/(14,10)^*b*^	(14,10)^*a*^	(14,10)	(12,9)
S_1_	4.38^*c*^	nπ* 4.93 (0.00)	nπ* 4.91	nπ* 4.85	nπ* 4.90	nπ* 5.18	nπ* 5.13	nπ* 4.83
S_2_	5.1^*d*^	ππ* 5.18 (0.20)	ππ* 5.09	ππ* 5.32	ππ* 5.23	ππ* 6.82	ππ* 7.04	ππ* 7.07
S_3_	6.0^*d*^	ππ* 6.18 (0.07)	nπ* 6.41	ππ* 6.02	ππ* 6.15	ππ* 7.29	nπ* 7.07	ππ* 7.33
T_1_	—	ππ* 3.80	ππ* 3.90	ππ* 3.90	—	ππ* 3.98	ππ* 4.00	ππ* 3.93
T_2_	—	nπ* 4.71	nπ* 4.84	nπ* 4.71	—	nπ* 4.87	nπ* 4.95	nπ* 4.65
T_3_	—	ππ* 5.33	ππ* 5.54	ππ* 5.47	—	ππ* 5.76	ππ* 5.86	ππ* 5.70

^*a*^Climent *et al.* ^29^

^*b*^For ππ*: SA5(A′)-CASPT2(10,8)/6-31G*, for nπ*: SA4(A′′)-CASPT2(14,10)/6-31G* from Schreiber *et al.* ^125^

^*c*^Masaaki Fujii and Ito.^128^

^*d*^Clark *et al.* ^124^

The critical analysis above illustrates that the choice of the level of theory for the dynamics can be very important, as the state energies are expected to influence the time evolution of the system and determine the population of the states. Particularly the energy gaps govern, together with the couplings, the hopping probabilities to other singlet states or to the triplet states, and therefore it seems natural to think that different levels of theory could deliver different results, distorting the interpretation of the experiments. Ideally, one would like to employ CASPT2 for the on-the-fly calculations since it is one of the most reliable *ab initio* methods in this case. Due to unfavorable scaling of CASPT2, we are limited to use CASSCF instead. Nevertheless, to assess the impact of the active space on the dynamics, the two active spaces ((14,10) and (12,9)) have been employed for the subsequent SHARC molecular dynamics calculations. The (14,10) active space has previously been used in the dynamical simulations of Fingerhut *et al.* ^104,105^ including singlets. The (12,9) active space is chosen since it predicts the same order of the states as CASPT2.^29,125,129^

**Fig. 3:**
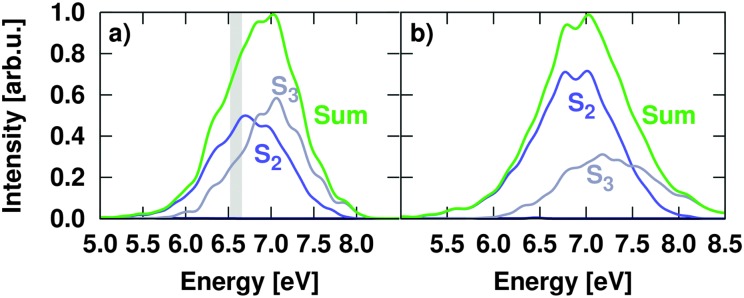
Simulated absorption spectrum of uracil using SA7-CASSCF(14,10) (in panel a) and SA7-CASSCF(12,9) (in panel b) single point calculations. Grey area denotes the excitation energy window employed to model the experimental excitation energy in uracil.

Using the 2000 uncorrelated geometries of the Wigner distribution, an absorption spectrum was calculated from the oscillator strengths and excitation energies. Fig. 3 shows the absorption spectra calculated with both active spaces. As it can be seen, the system is initially excited to the S_2_ and S_3_ states. Both states are bright as a direct result of the distribution of the initial geometries around the equilibrium geometry. Even if the S_3_ is a dark nπ* at the ground state equilibrium geometry using the (14,10) active space, a small geometrical displacement can alter the order of state characters, especially when both states are energetically close. Thus, S_3_ takes ππ* character and hosts a significant amount of excited population. As a result, irrespective of the initial character of the states, both S_2_ and S_3_ contribute to the absorption spectrum obtained with CASSCF. The relative distribution of population in the S_2_ and S_3_ states depends, however, on the active space; specifically, the S_2_ state dominates the spectrum in the energy region explored in our simulations with the (12,9) active space. Globally, the lack of dynamic correlation in the CASSCF method shifts this band towards higher excitation energies. Accordingly, the maximum of the simulated absorption spectrum is located at 176 nm (7.03 eV), overestimated by almost 2 eV, as in the papers of Nachtigallova *et al.* ^103^ using CASSCF(10,8) or Fingerhut *et al.* ^104,105^ using CASSCF(14,10).

Based on the obtained absorption spectra, four sets of trajectories were prepared (see Table 2). Ensemble I was taken as a reference set to assess the impact of ISC. Accordingly, only IC within the singlet states was allowed and any possible ISC towards the triplet states was neglected. This set contained 49 trajectories prepared at the CASSCF(14,10) level of theory, from which 26 and 23 were excited to the S_2_ and S_3_ states, respectively, according to the associated oscillator strengths. The trajectories were selected as to cover the full excitation range and were propagated during 600 fs. Ensemble II was composed of 120 trajectories from the CASSCF(14,10) initial conditions, also covering the full spectrum, and were split in 64 and 56 over the states S_2_ and S_3_. Since experimental setups do not cover the full spectrum but use a laser pulse of fixed wavelength, a third set of trajectories (Ensemble III) was prepared to match the energy range accessed experimentally. Gas phase pump–probe experiments in uracil^12–14^ typically employ a wavelength of 267 nm (4.64 eV) and pulse widths around 50 fs, resulting in an energy bandwidth of ±0.07 eV. Accordingly, the experimental excitation wavelength is about 0.44 eV below the observed absorption maximum of 224 nm (5.08 eV). Hence, to investigate the influence of the excitation energy on the excited state dynamics of uracil, Ensemble III was prepared from 64 trajectories, distributed as 37 and 27 in S_2_ and S_3_, respectively, resembling the distribution of states within the energy window of 0.14 eV centered around 6.59 eV. Finally, the influence of the active space is evaluated from the simulations of Ensemble IV, which comprises 40 trajectories, where CASSCF(12,9) was used in the dynamics instead of CASSCF(14,10). Energies were chosen to cover the full range of the theoretical absorption spectrum. From these trajectories, 28 and 12 were initially excited to the S_2_ and S_3_ states, respectively, and propagated for 500 fs.

**Table 2:** Ensembles of trajectories used in the dynamical simulations, with active space used, number of trajectories excited to S_2_ and S_3_, total number of trajectories propagated, number of states averaged in the CASSCF calculation, maximum propagation time and energy restrictions

	Ensemble			
	I	II	III	IV
CASSCF	(14,10)	(14,10)	(14,10)	(12,9)
S_2_ excitation	26	64	37	28
S_3_ excitation	23	56	27	12
Total	49	120	64	40
State averaging	SA4	SA7	SA7	SA7
*t*_max_ [fs]	600	1000	1000	500
Restrictions	—	—	6.52–6.66 eV	—

### 3.2 Excited state dynamics including singlet states only

**Fig. 4:**
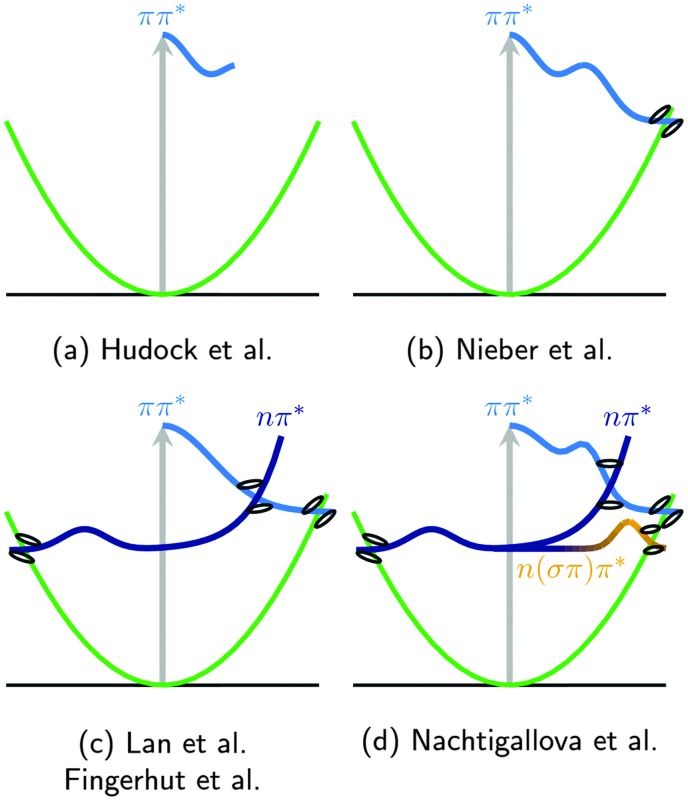
Schematic overview of the proposed deactivation mechanisms of uracil, from ref. 99 (a), ref. 100 (b), ref. 102, 104 and 105 (c), and ref. 103 (d). Note that the one-dimensional cartoons imply different reaction coordinates.

A number of gas phase excited-state dynamical studies including only singlet states are available for uracil. In Fig. 4 we have sketched the mechanisms derived from these studies.

Using CASSCF(8,6) wavefunctions and full multiple spawning (FMS), Hudock *et al.* ^99^ found in 2007 that after excitation uracil gets trapped in the S_2_ minimum (see Fig. 4a). One year later, Nieber, Doltsinis and coworkers^100,101^ employed surface-hopping trajectories coupled to Car–Parrinello dynamics on potentials calculated with the ROKS/BLYP approach and observed a sub-ps direct decay from the ππ* state to the ground state, governed by the so-called ethylenic ππ*/S_0_ CoIn (see Fig. 4b and also Section 3.4). The semiempirical-based OM2/MRCI simulations of Lan *et al.* ^102^ obtained two different relaxation mechanisms, as depicted in Fig. 4c. The first path directly connects the bright ππ* state with the S_0_ ground state *via* the ethylenic CoIn, while the second, slower pathway, connects the initially excited ππ* state with the nπ* state *via* a planar S_2_/S_1_ CoIn that is located close to the Franck–Condon region. The trajectories spend some time in the nπ* state until finally reaching the S_0_ *via* a different S_1_/S_0_ CoIn.

The surface-hopping simulations at the CASSCF(10,8) level of theory of Barbatti *et al.* ^24^ and by Nachtigallova *et al.*,^103^ showed that three deactivation pathways (which involve three electronic states) are possible – see Fig. 4d. The first one is equivalent to that suggested before in ref. 99–101: after initial trapping in the S_2_ minimum, the trajectories go to the S_0_ *via* a ππ*/S_0_ CoIn. Interestingly, this path is not observed in a comparable study made on thymine,^130^ even though thymine and uracil have very similar potential energy surfaces and analogous dynamics would be expected. The authors of ref. 24 and 103 argue that the efficiency of the direct ππ* → S_0_ path in thymine is significantly reduced due to the heavy mass of the methyl group. The second pathway found in uracil is also not observed in thymine and involves a crossing with the nπ* state. The implicated CoIn, termed ring-opening CoIn, leads to the destabilization of the ground state as the ring breaks. At these geometries the S_1_ wavefunction contains contributions of σ orbitals and therefore it is described as a σ(n–π)π* state. In their work, it is also noted that this pathway would probably lead to photochemical products different from the equilibrium geometry. Interestingly Buschhaus *et al.* ^131^ do observe ring opening after UV irradiation of nucleosides but the detected isocyanates (R–N=C=O) cannot arise directly from the N_3_–C_4_ bond cleavage predicted by Nachtigallova *et al.* ^103^ The third deactivation pathway involves a change of character to nπ* after initial trapping and relaxation through the S_2_/S_1_ CoIn. The trapping in the nπ* minimum delays the ground state relaxation, affecting the time scales obtained.

The most recent surface-hopping simulations have been published by Fingerhut and coworkers^104,105^ and are based on CASSCF(14,10) wavefunctions that describe four singlet states. After initial excitation a fast decay of the S_3_ population is observed (not shown in Fig. 4c), together with a slower decrease of S_2_ population and an increase of S_1_ population that exceeded the S_2_ population after about 400 fs of simulation time, in agreement with the two mechanisms of Lan *et al.* ^102^ In the first pathway, the initially populated S_2_ state of ππ* character decays to the S_1_(nπ*) state, gaining more than 20% of population in less than 100 fs. In the nπ* state, population can be trapped before decaying to the S_0_, leading to long relaxation times. Interestingly, this study shows much less pronounced trapping in the S_2_ state than the one of Nachtigallova *et al.*,^103^ even though both studies use CASSCF. Fingerhut *et al.* ^104^ attribute the difference to the size of the active space size. The second pathway involves a transition to S_1_ without changing the state character (*i.e.* staying in the ππ* state), followed by ππ* → S_0_ relaxation through the ethylenic CoIn. In their simulations, all trajectories that reached the ground state within 1 ps followed the second pathway and only a few trajectories relaxed *via* the first pathway in longer runs of up to 2 ps.

**Fig. 5:**
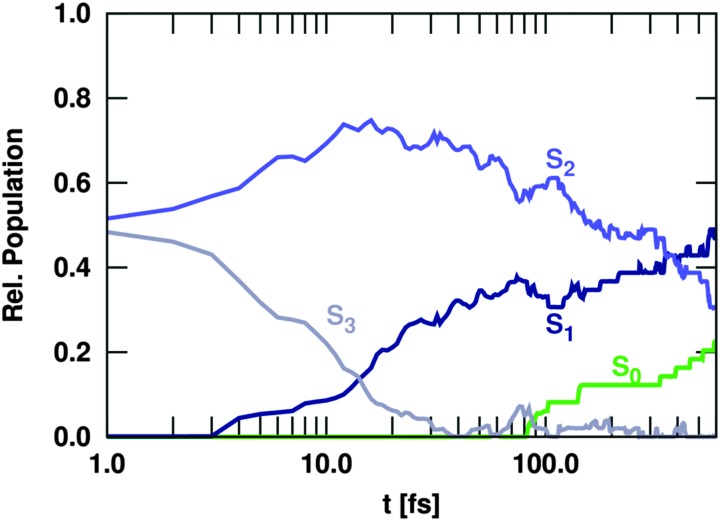
Time evolution of the population of electronic states, including only singlet states (Ensemble I).

All the previous simulations can be compared with the results obtained using the Ensemble I of trajectories. Fig. 5 shows the time evolution of the different state populations. Not surprisingly, our results are comparable to that of Fingerhut *et al.* ^104^ because they are made at a similar level of theory. In essence, the S_2_(ππ*) state decays to the S_1_(nπ*) and repopulation of the ground state occurs within 100 fs. As it will be shown in the next section, the inclusion of triplet states changes this picture dramatically.

### 3.3 Excited state dynamics including singlet and triplet states

**Fig. 6:**
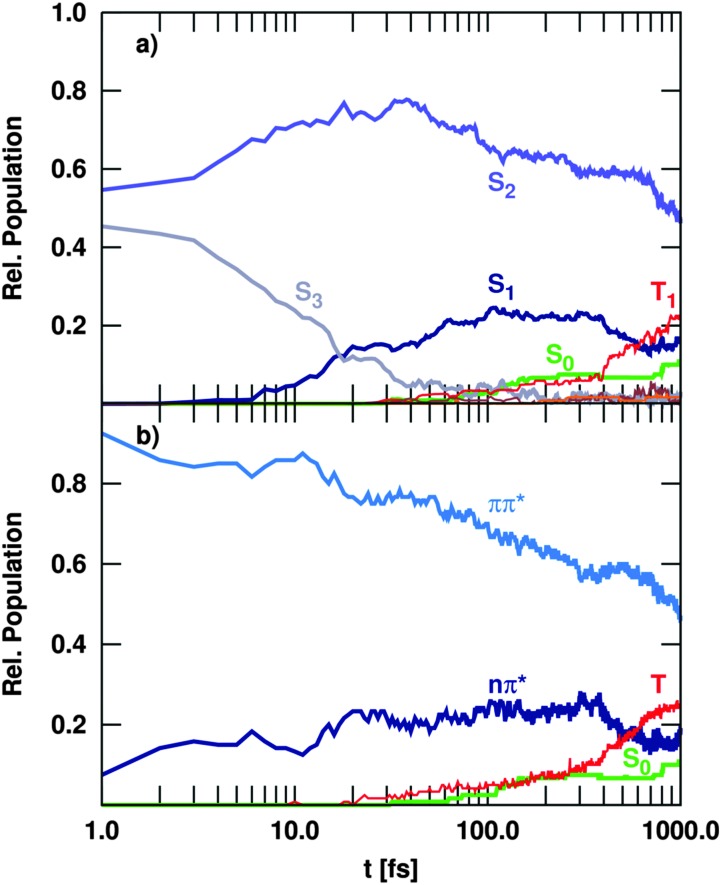
Time evolution of the population of electronic states, including singlet and triplet states (Ensemble II), in the MCH (a) and spectroscopic (b) representation.

Fig. 6a shows the time-evolution of the singlet and triplet state populations of the trajectories considered in the Ensemble II (recall Table 2). Although the propagations are made in the basis of fully diagonal, spin-mixed states, the analysis is carried out in the MCH and spectroscopic representations in order to be able to compare to previous studies.

As in the singlet-only case (Ensemble I), the trajectories initially excited to the S_3_ state show a very efficient decay to S_2_. Therefore, after 200 fs the S_3_ is completely depopulated and after this time, this state plays a negligible role in the deactivation of uracil. This ultrafast IC from the S_3_ generates substantial amount of population in the S_2_ state, which within the first 40 fs collects about 75% of the total population – in similitude to the singlet-only case (Fig. 5). During the rest of the propagation time, the population of the S_2_ state decreases slowly due to IC to the S_1_. However, after 1 ps still 50% of the excited trajectories are trapped in the S_2_, indicating that the population resides in the surrounding of the S_2_ minimum. The rest of the population is transferred to the S_1_, which gains some population in the beginning from S_3_ and later on slowly *via* S_2_ → S_1_ transitions, but not as substantially as in Fig. 5. From the S_1_, some trajectories decay to the S_0_ within the first 200 fs *via* the so-called ring opening CoIn (see Fig. 10c in the next section). Only after this time, the S_0_ population starts increasing very slowly, unlike in the singlet-only case. The reason is obviously that there is a significant amount of population accumulated in the triplet states after the total propagated time. With 22%, the T_1_ state has the largest population, after the S_2_ with 50%, whereas the S_1_ accumulates up to 15% and other higher excited states (S_3_, T_2_, T_3_) show only populations below 3%. The T_1_ state is mostly populated *via* S_1_ → T_2_ ISC, followed by very efficient T_2_ → T_1_ IC. This qualitative distribution of populations is fundamentally different from the case where only transitions within the singlet manifold are allowed (Ensemble I shown in Fig. 5) because an additional channel (ISC) has been opened to compete dynamically with IC.

**Fig. 7:**
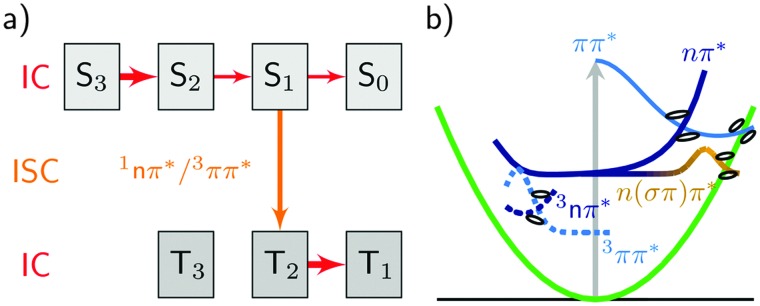
Overview of the processes observed in uracil. (a) MCH representation: the thickness of the arrows indicates the extent of population transfer. IC and ISC stand for internal conversion and intersystem crossing, respectively. (b) Spectroscopic representation: although one-dimensional, the plot implies multiple reaction coordinates.

Neglecting the possibility of ISC in the simulations results in a strong increase of nπ*/S_1_ population. Thus, the population of the singlet nπ* state exceeds the population of the ππ*/S_2_ state after about 400 fs (Fig. 5). This population inversion is not observed in the simulations that allow for population of the triplet states *via* ISC (Fig. 6a). Here, the depopulation of S_1_ due to ISC processes prevents the population inversion of the excited singlet states as well as the efficient population of the S_0_ ground state within 1 ps. Thus, our simulations suggest that ISC processes in uracil are too slow to compete with IC of the excited singlet states in the early times, but can significantly alter the fate of the population trapped in the lowest excited singlet state. As a result, IC to the ground state and ISC to the triplet manifold are in direct concurrence. A general decay scheme summarising the processes discussed above is given in Fig. 7a.

The majority of the S_1_ → T_2_ transitions follow El-Sayed's rule,^132,133^ *i.e.* the transitions involve a change of state character in the singlet–triplet transition. Usually the S_1_ is of nπ* character and the T_2_ is of ππ* character (see ESI† on how to evaluate the state character), resulting in large SOCs that peak above 60 cm^–1^ (39 cm^–1^ on average). The energy difference of the involved states at the ISC geometries is very small, 0.01 eV (98 cm^–1^) on average, favouring the process of ISC.^31^

**Fig. 8:**
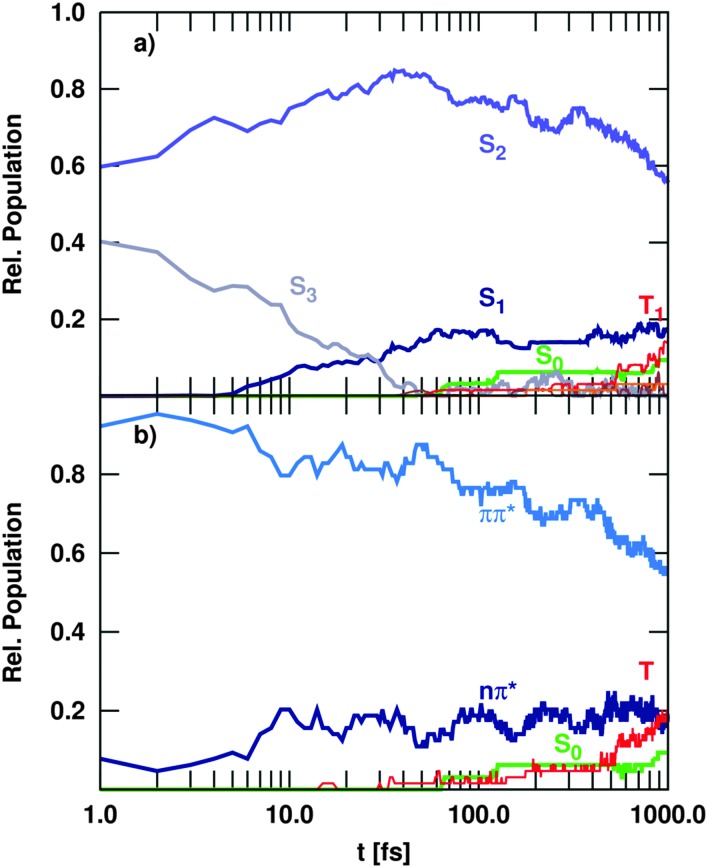
Time evolution of the population of (a) electronic states and (b) spectroscopic states, including singlet and triplet states excited within the energy range to 6.59 ± 0.07 eV (Ensemble III).

Restricting the allowed excitation energy range to 6.59 ± 0.07 eV (Ensemble III) yields qualitatively similar results, see Fig. 8. However, the energetic restriction leads to a smaller triplet yield and a concomitantly slightly larger population in the ground state. This behaviour results from the smaller total energy of the trajectories which reduces the probability to reach geometries that allow for efficient ISC, giving the system more time to relax *via* S_1_/S_0_ CoIns to the electronic ground state.

For a more detailed comparison of the dynamical simulations with the experiment, it is useful to follow the time evolution not of the MCH states (S_0_, S_1_, *etc.*) but of the spectroscopic states, *i.e.* the states classified according to their character (nπ*, ππ*, *etc.*).^122^ Indeed, time-dependent experiments do not monitor occupations in states ordered by energy, but follow the change of physical properties, such as oscillator strengths or ionization yields, that heavily depend on the character of the states. Therefore, we show the spectroscopic populations in Fig. 6b for Ensemble II (the results for Ensemble III are qualitatively very similar). The comparison between both representations (panels a and b) shows a strong correlation of the S_2_ state with the ππ* character and the S_1_ with nπ*. Initially, all trajectories start in a bright ππ* state that corresponds to S_2_ and S_3_, recall Fig. 3a. The decay of the S_3_ population to the S_2_ in the MCH picture hence corresponds to vibrational relaxation in the bright ^1^ππ* state. A sketch in Fig. 7b illustrates this pathway, where the ^1^ππ* state is indicated as solid, lightblue curve. Along this path, IC to the dark nπ* state (indicated as solid, dark blue curve) can occur. This ^1^nπ* state mostly corresponds to the S_1_ state in the MCH representation. A branching can lead further to the ring-opening discussed above (n(σπ)π*, solid yellow). More importantly, the nπ* is the doorway for ISC to the triplet states. ISC then leads to the ^3^ππ* state (dashed, light blue), where vibrational relaxation towards the ^3^ππ* minimum takes place (implying an IC from T_2_ to T_1_ in the MCH picture).

**Fig. 9:**
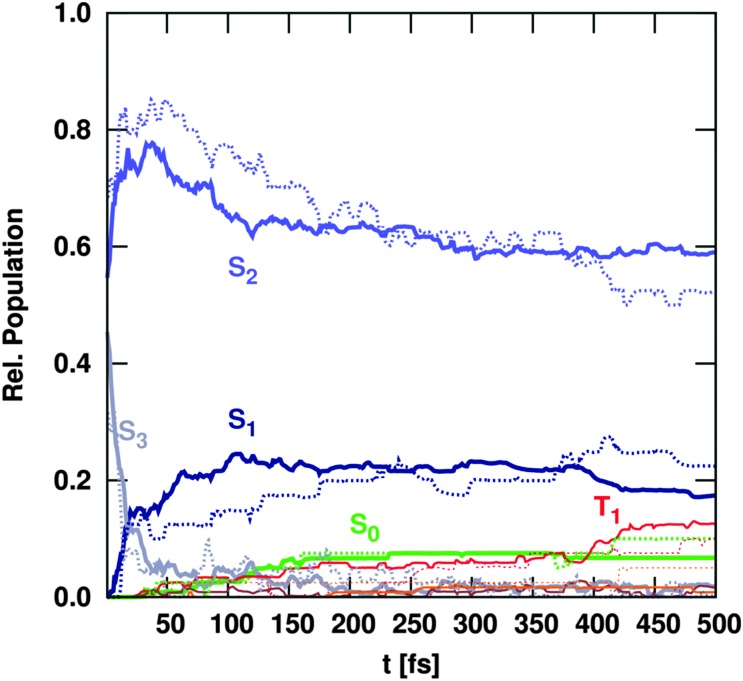
Comparative time evolution of electronic state populations using the CASSCF(14,10) (Ensemble II, straight lines) and CASSCF(12,9) (Ensemble IV, dotted lines) level of theory.

As was shown before in Fig. 3 and Table 1, a change of the active space of a CASSCF calculation leads to a change of the potential energy surfaces, which might lead to different dynamics. The impact of the active space onto the relaxation dynamics has been investigated with the dynamical simulations of Ensemble IV. Fig. 9 shows an overlay of the electronic state populations of Ensembles II (CASSCF(14,10)) and IV (CASSCF(12,9)) within the first 500 fs after *δ*-pulse excitation. Obviously, the initial populations mirror the Wigner distribution of geometries, so that Ensemble IV shows a higher population of S_2_ in the first 200 fs. Also the initial relaxation from S_2_ to S_1_ is slower in Ensemble IV, as a result of the increased energy gap between those states at the CASSCF(12,9) level of theory in comparison to CASSCF(14,10) energies (see Table 1). After 200 fs, however, the differences between the electronic state populations are not substantial, suggesting that qualitatively the effect of the active space is not dramatic in this case. The differences in the approximate spectroscopic states (not shown) are even smaller, showing that the results are robust with respect to different active spaces with different state ordering at the Franck–Condon geometry (see Table 1).

### 3.4 Conical intersections and hopping structures

**Fig. 10:**
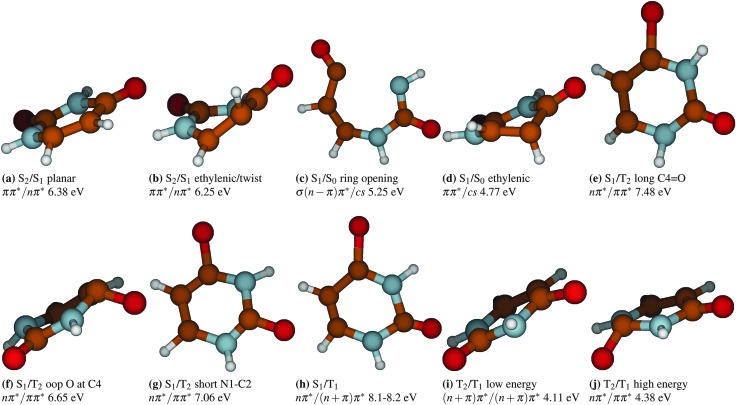
Exemplary structures mediating intersystem crossing and optimized structures that mediate internal conversion in uracil.

It is well-known that the population transfer in molecular dynamics simulations does not exactly happen at the minimum energy points of the seams of CoIns.^134^ One can, however, relate the hopping geometries with the CoIns that can be optimized by means of static quantum chemical calculations. To this aim, all the hopping geometries where IC between singlets and IC between triplets occurred have been used as a starting point of a state crossing optimization. Some of the obtained geometries correspond to previously reported geometries, others are new crossing points which have been discovered by the dynamics. Fig. 10 collects all the geometries optimized in this work as well as exemplary geometries that illustrate frequently observed features of ISC transitions.

Fig. 10a corresponds to a ππ*/nπ* CoIn that allows for S_2_/S_1_ hops. This structure has been previously found by Merchán *et al.* ^135^ and Lan *et al.* ^102^ and predicted at 5.92 eV above the equilibrium ground state energy at CASSCF level of theory. In our calculations, this structure is higher in energy (6.38 eV) due to the larger number of averaged states. One hop from S_2_ and S_1_ was found mediated by a different structure, depicted in Fig. 10b, which is characterized by a twist of the ethylenic HC–CH group and a strong out-of-plane distortion of the aromatic ring. As already stated by Lan *et al.*,^102^ this reduction of planarity lowers the energy, in this case by 0.13 eV (CASSCF). However, precisely due to the strong distortion it is unlikely for many trajectories to proceed to this specific region of the potential energy surface (as corroborated by the present simulations where only one hop was found facilitated by this structure).

In the deactivation from S_1_ to S_0_, two geometries have been located. The most frequently used by uracil is the ring opening CoIn depicted in Fig. 10c where the aromatic ring of the system is opened to maintain the planarity. The second CoIn, shown in Fig. 10d, involves a twist of the ethylenic bond and it is therefore often referred to as the ethylenic S_1_/S_0_ CoIn. This latter structure was observed only in 3 hopping events. Both CoIns have been also reported by Nachtigallova *et al.* ^103^ Thus, it can be concluded that the deactivation to the ground state within the first 200 fs is mediated by the ring opening CoIn and later deactivation happens *via* the ethylenic CoIn. These two mechanisms contribute to explain the biexponential increase behaviour that can be observed when fitting the populations of the S_0_ and T_1_ (*vide infra*).

ISC is mainly mediated by S_1_ → T_2_ transitions. Several structures could be obtained where these states come close in energy (<1 meV). These structures are dominated by heavy changes of the C=O bond lengths (Fig. 10e) or ring puckering (Fig. 10f). Ring deformations, such as the reduction of the N1–C2 bond length to only 1.22 Å can also lead to degeneracy between both states (Fig. 10g).

In contrast to the S_1_ → T_2_ transitions, S_1_ → T_1_ ISC occurs rarely and only one hopping structure has been found where both states are energetically close to each other (Δ*E* < 0.1 eV). This structure, see Fig. 10h, is characterized by a strongly elongated C4=O bond (1.70 Å) and a deformed aromatic ring with a short C5–C6 bond (1.27 Å) and a long C4–C5 bond (1.55 Å).

IC between the T_2_ and T_1_ states is facilitated by two different geometries that are about 4.11 and 4.38 eV above the ground state equilibrium energy at CASSCF level of theory. The geometry lower in energy shows an out-of-plane displacement of the oxygen at C4 by about 21°. The C4=O bond is elongated to about 1.37 Å, whereas the C2=O bond is shortened to 1.20 Å (Fig. 10i). This structure is very similar to the one described by Climent *et al.* ^29^ using quantum chemical calculations. The other CoIn lies 0.3 eV higher and shows the oxygen at C2 sticking out of the ring plane by about 54° and the C2=O bond elongated to 1.41 Å (Fig. 10j).

### 3.5 Decay times

Uracil has been subject of a number of time-dependent experimental studies, whose reported time constants and experimental setups are collected in Table 3. The first pump–probe experiments in gas phase in uracil were made by Kang *et al.* ^16^ Using a pump pulse of 267 nm excitation and multi-photon (*n* × 800 nm) ionization as a probe, a monoexponential decay of the ionization signal yield with a time constant of 2.4 ps was fitted. Using higher time resolution, later studies were able to find an additional ultrashort time constant between 50 and 130 fs. Interestingly, except for the time-resolved photoelectron spectra of Ullrich and coworkers,^15^ recorded with a 250 nm excitation and a 200 nm probe pulse, who fitted a 3-exponential decay (<50 fs, 530 fs, 2.4 ps), most experiments find a biexponential decay behaviour after photoexcitation. In 2005, Canuel *et al.* ^14^ observed a decay of the transient ionization signal with time constants of 130 fs and 1.1 ps and the fluorescence upconversion experiments of Gustavsson *et al.* ^17,18^ recorded an ultrafast decay of fluorescence in aqueous solution with a time constant of less than 100 fs. Recent experiments of Kotur *et al.* ^12^ and Matsika *et al.* ^13^ combine TOF-MS with strong field ionization (*n* × 780 nm) to obtain insight into the differences between the dynamics of different uracil fragments and the parent ion. In all their studies, they report a biexponential decay with a short time constant of 70–90 fs and a long time constant in the picosecond region (2.2–3.2 ps).

**Table 3:** Decay times of uracil as measured by pump–probe experiments in gas phase or solution (denoted by^*a*^) or calculated theoretically using different methods, as indicated. The symbol X indicates that the relevant paper discusses that timescale without giving a quantitative time constant. Numbers in parentheses in the third block refer to Ensemble III (see text)

Setup		*τ*_1_ [fs]	*τ*_2_ [fs]	*τ*_3_ [ps]	Ref.
**Experiment**					
*λ*_pump_ [nm]	*λ*_probe_ [nm]				
267	*n* × 800	—	—	2.4	Kang *et al.* ^16^
250	200	<50	530	2.4	Ullrich *et al.* ^15^
267	2 × 400	130	—	1.1	Canuel *et al.* ^14^
267	330^*a*^	96	—	—	Gustavsson *et al.* ^17^
267	330^*a*^	<100	—	—	Gustavsson *et al.* ^18^
262	*n* × 780	70	—	2.2	Kotur *et al.* ^12^ (parent ion)
262	*n* × 780	90	—	3.2	Kotur *et al.* ^12^ (69+ fragment)
262	*n* × 780	70	—	2.4	Matsika *et al.* ^13^ (parent ion)
262	*n* × 780	90	—	2.6	Matsika *et al.* ^13^ (69+ fragment)

**Theoretical method**					
FMS:CASSCF(8,6)		X	—	—	Hudock *et al.* ^99^
SH:CPMD/BLYP		—	551–608	—	Nieber *et al.* ^100^
					Doltsinis *et al.* ^101^
SH:OM2/MRCI		21	570	—	Lan *et al.* ^102^
SH:CAS(10,8)		—	650–740	>1.5–1.8	Barbatti *et al.* ^24^
					Nachtigallova *et al.* ^103^
SH:CAS(14,10)		X	X		Fingerhut *et al.* ^104^
SH:CAS(14,10)		—	516		Fingerhut *et al.* ^105^

**This work**					
S_0_ + T_1_		—	—	2.4 ± 0.1 (4.2 ± 0.1)	Ensemble II (III)
S_0_ + T_1_		63 ± 7 (48 ± 11)	—	2.8 ± 0.1 (5.2 ± 0.1)	Ensemble II (III)
ππ*		30 ± 1 (8 ± 1)	—	3.2 ± 0.1 (2.6 ± 0.1)	Ensemble II (III)

^*a*^Fluorescence upconversion in aqueous solution.

The time constants reported in previous theoretical studies are also listed in Table 3. Curiously, most theoretical studies have obtained mono- or biexponential decays, where the time constants have mostly been assigned to the intermediate transient observed by Ullrich *et al.* ^15^ Early FMS investigations of uracil by Hudock *et al.* ^99^ at the SA-CASSCF(8,6) level of theory report only small yields for IC within the first 500 fs after starting in the bright S_2_ state. They claimed the experimentally observed ultrafast time constant (*τ*_1_ in Table 3) to be the result of the system's initial vibrational relaxation towards the S_2_ minimum, hand-in-hand with a significant increase of the ionization potential. In contrast to this, the Car–Parrinello molecular dynamics studies of Nieber *et al.* ^100^ and Doltsinis *et al.* ^101^ employing the BLYP functional, observe a single time constant of 608 fs for the S_1_ → S_0_ deactivation at 300 K that decreases to 551 fs at 0 K temperature. The semiempirical surface-hopping simulations of Lan *et al.* ^102^ describe a biexponential decay with time constants of 21 and 530 fs, attributing the fast component to the S_2_ → S_1_ transitions and the slow component to the relaxation from S_1_ to S_0_. The *ab initio* surface-hopping simulations reported by Barbatti *et al.* ^24^ and Nachtigallova *et al.* ^103^ also find a biexponential decay. Depending on the initial energy of the trajectories, to match a 250 nm or 267 nm excitation, they arrive at time constants of 650 fs and >1.5 ps or 740 fs and >1.8 ps, respectively. The faster of the two time constants is attributed to a relaxation to the ground state by either ππ*/S_0_ decay after being trapped in the ππ* minimum or a decay *via* an opening of the aromatic ring, leading to a mixed σ(n–π)π* character of the excited state when the transition to the ground state occurs. The longer time constant is attributed to the trapping of the trajectories in the dark nπ* state before they relax to the ground state. In the recent study of Fingerhut *et al.*,^104^ explicit lifetimes are not given, but a fast S_2_(ππ*) → S_1_(nπ*) decay is observed as well, leading to a population of more than 20% of the initially dark S_1_(nπ*) state after less than 100 fs. In this state, the trajectories get trapped before they slowly proceed towards the S_0_ ground state. A second study of Fingerhut *et al.* ^105^ using a larger ensemble of trajectories allowed them to extract a time constant of 516 fs for the depopulation of the bright S_2_(ππ*) state.

The third block of Table 3 collects time scales fitted in this work from Ensemble II, values from Ensemble III are given in parentheses. As a prerequisite for fitting time constants that are comparable to experimental results, the neutral states leading to the experimental signal need to be identified. In case of an ionization setup, states with an ionization potential larger than the multiphoton ionization energy (*i.e.* states of the neutral molecule much lower in energy than the ground state of the ion) are assumed as dark, while all other states exhibit different brightnesses. It is often assumed that only the S_0_ ground state is dark in these studies and the decay constant fits are based on this state's population. However, it has been shown for cytosine, that additionally the lowest triplet state (T_1_), may be dark for the probe pulses typically employed in the experiments.^36^ Since cytosine and uracil are structurally very similar and the quantum yield of S_0_ obtained in our simulations is low, fitting was performed over the sum of S_0_ and T_1_ population, analogously to cytosine. Note that fitting ought to be carried out in the spectroscopic representation, but in our case, the S_0_ coincides with the spectroscopic closed-shell state. Moreover, triplets cannot be distinguished in our way of determining the spectroscopic states, but we should include only the lowest triplet state in the fit. Thus, it is justified exceptionally to perform this part of the analysis using the S_0_ and T_1_ population. Fitting of the S_0_ and T_1_ population of Ensemble II with a biexponential function yields two time constants of 63 fs and 2.8 ps.

The fs time constant stems from an early relaxation of population to S_0_ and T_1_ *via* the ring-opening CoIn (see Fig. 10c). However, static calculations at higher level of theory predict a strong destabilization of the ring-opening CoIn^103^ and this pathway might actually be blocked. Therefore, a monoexponential fit was performed in addition, which yields a lifetime of 2.4 ps in good agreement with the experimentally observed ps time constant. Note however that any time constants in the range of several picoseconds have to be taken with a grain of salt since the simulation time was restricted to 1 ps. Also, the errors given in Table 3 represent the asymptotic standard errors of the fitting procedure alone and do not take into account further errors introduced by other approximations included in the simulation. Therefore, they do not represent errors with respect to real, experimentally measurable decay times. The mechanism behind the ps time constant involves several processes. Population relaxes from the ππ* to the nπ* state *via* the CoIns shown in Fig. 10a and b. From the nπ* state, ISC leads mostly to the triplet T_2_ state of ππ* character in this region (exemplary structures are shown in Fig. 10e–h) followed by ultrafast IC to the lowest triplet state T_1_ (ππ* in this region). Population in the ^1^nπ* that does not deactivate *via* ISC, can reach the S_0_ ground state *via* the CoIns shown in Fig. 10c and d. Since the monoexponential fit describes the population decay well, it can be followed that ISC and ground state relaxation occur on comparable timescales.

So far, only the decay to states that are assumed completely dark has been considered in the fitting procedure. However, the experimental signal may be also changed by transitions to states where the brightness is different from the state occupied before but non-zero. This is the case in uracil for the transition from the ππ* to the nπ* state.^136,137^ The ππ* state is quickly depopulated during the first few fs and afterwards the decay slows down considerably. Consequently, the depopulation of the ππ* state was fitted biexponentially, yielding a time constant of 30 fs for the initial ππ* to nπ* decay. This relaxation proceeds *via* the CoIns shown in Fig. 10a and b, as already discussed for the ps time constant. However, the pathway here is slightly different. Initially, population is excited to the ππ* state in the Franck–Condon region. From there, the ensemble of trajectories moves almost coherently towards the potential minimum of the ^1^ππ* state and a fraction relaxes *via* said CoIns to the ^1^nπ*. Especially the planar structure shown in Fig. 10a is similar to the equilibrium structure of ground state uracil and therefore easily accessible from the Franck–Condon region. This fast decay of the bright ^1^ππ* state population is also in qualitative agreement with the fast decay of fluorescence in the experiments of Gustavsson *et al.* in solution.^17,18^ The trajectories remaining in the ^1^ππ* state proceed towards the ^1^ππ* minimum and only at later times the trajectories can move back to the ^1^ππ*/^1^nπ* CoIns. Since this return requires then a motion up in potential energy, the process is slowed down, leading to time constant of 3.2 ps. This constant is very similar to the one for the decay to S_0_ and T_1_, but slightly slower. Hence, the nπ* state is slower replenished than depopulated, leading to a slight decay of its population.

Restricting the excitation energy to a small window below the absorption maximum (Ensemble III) increases the lifetimes of the monoexponential fit of S_0_ and T_1_ population to 4.2 ps and for the biexponential fit to 48 fs and 5.2 ps in agreement with the results of Nachtigallova *et al.* ^103^ The biexponential decay of ^1^ππ* population in Ensemble III yields time constants of 8 fs and 2.6 ps. The first constant is even faster than in Ensemble II and hence also below the time resolution of the discussed experimental studies. The second constant is shortened as well and is now faster than the S_0_ + T_1_ time constant in the ps regime. As a consequence, the population of the nπ* state remains more or less constant after an initial steep rise. This behaviour is in line with a summation of the seemingly different time constants of 8 fs, 2.6 ps (ππ* depopulation) and 4.2 ps (S_0_ + T_1_ population). Note however that a multitude of different processes underly the found time constants including electronic transitions through various CoIns, vibrational relaxation and ISC. To find the connection between experimental time constants and the underlying physical processes is hence extremely challenging without the help of theoretical predictions. For a detailed discussion of the fitting functions used to obtain the presented time constants, the interested reader is referred to the ESI.†

Interestingly, we do not find the intermediate time constant that was reported experimentally only once in ref. 15 and could not be reproduced in later experimental studies. Theoretical studies assign this time constant to different processes, ranging from the S_0_ population obtained from DFT and CASSCF studies with small active spaces^24,100,101,103^ to the depopulation of the bright S_2_/ππ* state obtained using CASSCF with larger active spaces.^105^ Thus, the origin of this time constant remains unclear.

Generally, the existence of an ultrafast time constant below 100 fs and a long time constant of several ps is in agreement with recent experimental studies^12–14^ (see Table 3). The short time constant is somewhat smaller than experiments suggest, what could be a result of the limited experimental time resolution and the theoretically employed *δ*-pulse excitation. The long time constant however is in excellent agreement with experimental results, confirming the validity of our findings.

## 4 Conclusions

In summary, this study provides clear evidence that intersystem crossing should be considered in any time-dependent treatment of the relaxation processes in uracil. Whereas time-independent quantum chemical computations can provide important intermediate structures of the potential energy surfaces, like conical intersections, minima and transition states, time-dependent dynamical studies are mandatory to evaluate the impact of these structures on the actual relaxation mechanism. As shown in this study, the exclusion of possible interactions between states of different multiplicity can qualitatively change the photophysical picture of the processes taking place.

Our dynamical calculations including both non-adiabatic and spin–orbit couplings show that the deactivation mechanism of uracil after UV light irradiation is the result of several competing processes. After 1 ps a significant fraction of the population can be found in the S_2_, which mainly corresponds to the ππ* state. The relaxation process can be characterized by a biexponential decay. A fast component *τ*_1_ (30 fs) is attributed to the change of state character from the initially excited ππ*. The slower constant *τ*_3_ (2.4 ps) arises from intersystem crossing in direct competition to internal conversion. The S_1_ state, which is of nπ* character, was found to be the doorway to triplet states, since population is trapped there for a sufficiently long time to allow intersystem crossing. In contrast to previous studies,^104,105^ only a very small amount of population returns to the ground state within 1 ps because ground state relaxation is quenched by intersystem crossing. The ground state relaxation is mediated by the ethylenic CoIn and the ring-opening path previously observed by Nachtigallova *et al.* ^103^

The direct comparison of the decay lifetimes to those experimentally detected yields very good agreement and therefore supports the conclusion, that intersystem crossing from the first excited singlet state is in direct concurrence to internal conversion towards the ground state of uracil. Solvent effects could affect the observed time scales and the relative quantum yields of the involved processes. Therefore, the particular influence of solvation on intersystem crossing should be investigated in the future.
