# Supplementary material for: Ultrafast intersystem crossing dynamics in uracil unravelled by ab initio molecular dynamics
Source: Phys Chem Chem Phys. 2014 Oct 10;16(44):24423–36. doi: 10.1039/c4cp04158e (PMC4391640; doi:10.1039/c4cp04158e)
Supplement: Supplementary file 1 [file CP-016-C4CP04158E-s001.pdf]

Electronic Supplementary Material (ESI) for Physical Chemistry Chemical Physics.
This journal is © the Owner Societies 2014

Supplementary Information: Ultrafast Intersystem Crossing Dynamics in Uracil Unravelled by Ab Initio Molecular Dynamics†

Martin Richter, Sebastian Mai, Philipp Marquetand*, and Leticia González

Fitting of the Decay Constants

The decay time constants shown in Table 3 have been fitted using exponential functions. The monoexponential fit of the S_0 and T_1 populations has been done using the following formula:

$$P_{\mathrm{mono},S_0/T_1} = 1 - e^{\frac{-t}{\tau_1}} \quad (1)$$

The biexponential fit has been performed using the following formula:

$$P_{\mathrm{bi},S_0/T_1} = 1 - N \cdot e^{\frac{-t}{\tau_1}} - (1-N) \cdot e^{\frac{-t}{\tau_2}} \quad , \quad (2)$$

with N defining the ratio of population decaying with time-constant τ_1.

The decrease of $\pi\pi^*$ population was fitted using the following biexponential function:

$$P_{\pi\pi^*} = N \cdot e^{\frac{-1}{\tau_1}} + (1-N) \cdot e^{\frac{-t}{\tau_2}} \quad , \quad (3)$$

with N defining the ratio of population decaying with time-constant τ_1.

A summary of all fitting constants is given in Table SI1

Table SI1 Constants used during the fitting of lifetimes of the excited states of uracil.

Ensemble	State(s)	N	τ_1 [fs]	τ_2 [fs]
II	S_0/T_1		2387 ± 8	
II	S_0/T_1	0.0416 ± 0.0016	63 ± 7	2847 ± 22
II	$\pi\pi^*$	0.3274 ± 0.0020	30 ± 1	3213 ± 53
III	S_0/T_1		4227 ± 25	
III	S_0/T_1	0.0293 ± 0.0019	48 ± 11	5248 ± 86
III	$\pi\pi^*$	0.1964 ± 0.0018	8 ± 1	2564 ± 27

Evaluation of State Character

The character of the S_1/T_2 hopping geometries as shown in Figure SI1 was calculated by determining the amount of contribution of basis functions located at the oxygen atoms of uracil and other atoms to the active orbitals. A threshold of 50% was applied to differentiate between an n and a π/π^* orbital. Afterwards, the $n\pi^*$ character of a given state was calculated from the CI coefficients for all configurations that resemble an $n\pi^*$ excitation. Configurations involving the excitation of several electrons were weighted only partly in case they also involved $\pi\pi^*$ excitations.

Institute of Theoretical Chemistry, Währinger Str. 17, 1090 Vienna, Austria, E-mail: philipp.marquetand@univie.ac.at

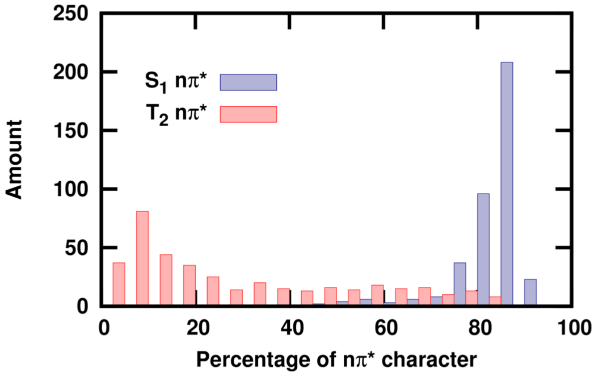

Fig. SI1 Percentage of nπ^* character of the S_1 and T_2 states at the trajectories hopping geometries.

Structures of Degeneracies and Conical Intersections

Figure SI2a shows the structure of the S_2 minimum that shows a slight bending of the C4=O bond and a twist of the C5=C6 double bond by 35°. The S_2 state is of $\pi\pi^*$ character at this geometry. Figures SI2b and SI2c show further characteristic deformations that lead to $S_1 \rightarrow T_2$ transitions, similar to those shown in Figure 10.

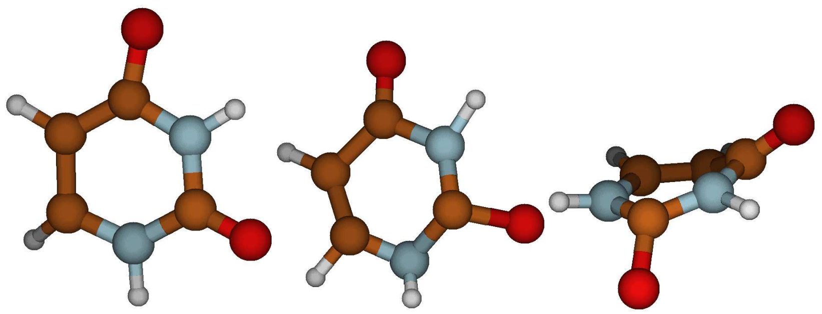

(**a**) S_2 minimum $\pi\pi^*$ 6.10 eV (**b**) S_1/T_2 short C4=O $n\pi^*/\pi\pi^*$ 7.74 eV (**c**) S_1/T_2 oop O at C2 $n\pi^*/(n+\pi)\pi^*$ 7.03 eV

Fig. SI2 Hopping geometries where intersystem crossing occurred and S_2 minimum.

Table SI2 S_2/S_1 planar structure

C	0.7967212453	-0.0280301979	-1.2071382112
C	1.4517341779	0.1012369344	0.1217244735
C	-0.5724707897	-0.0221614792	-1.2368639933
C	-0.7504279119	-0.0959085385	1.1913794609
N	0.6012557846	0.0681464962	1.2101861859
N	-1.2872425539	-0.0766844651	-0.0860996226
O	-1.3922484407	-0.0084333215	-2.3037524707
O	-1.4375686809	-0.2084183329	2.1727637907
H	-2.2827409095	-0.0940349516	-0.1529556493
H	2.3269732293	0.7124067528	0.2606681141
H	1.3702542326	-0.0271992266	-2.1081978724
H	0.9830459640	0.1475766133	2.1278974254

Table SI3 S_2/S_1 planar structure

C	0.8284922283	0.0863838810	-1.1640888422
C	1.4635244352	0.1853862509	0.1037481519
C	-0.5445468104	0.0921399570	-1.3867655395
C	-0.7196147463	0.0072790804	1.0863862235
N	0.5804502260	0.0098426373	1.2399885831
N	-1.2869375565	0.0892589284	-0.0710634756
O	-1.2665569285	0.0624632702	-2.3483404445
O	-1.4845146039	-0.0737609318	2.1905787900
H	-2.2835261885	0.0321686993	-0.1342682428
H	2.4363098056	-0.2335484734	0.2770274459
H	1.4511920663	0.0563967232	-2.0360881222
H	0.9377127424	0.0281312502	2.1697517770

Table SI4 S_2/S_1 twist structure

C	0.7918102149	0.1250536784	-1.1701701408
C	1.3867807898	-0.2211007821	0.1700155726
C	-0.5844248075	-0.0379705395	-1.2461199323
C	-0.7405096295	0.0616893509	1.1748177523
N	0.6754631123	0.1929268371	1.1987652937
N	-1.2075349527	-0.4308314672	-0.0040831232
O	-1.3647828298	0.2136071167	-2.1580645181
O	-1.3703536417	0.3045625548	2.1670628650
H	-2.2014449144	-0.5306700693	-0.0415548419
H	2.4183579086	-0.4613445740	0.3367900260
H	1.1517675197	1.0770728420	-1.5358948811
H	1.0535546005	0.3622642261	2.1126173680

Table SI5 T_2/T_1 low energy structure

C	0.8668653188	0.0532843719	-1.1450930076
C	1.4504641231	0.1860904574	0.0962881060
C	-0.5052505705	-0.1508396389	-1.1991336481
C	-0.7330302273	0.0839426027	1.2077802706
N	0.6287674593	0.2178145971	1.2313483070
N	-1.2959314406	0.0026832917	-0.0559315572
O	-1.2629725893	-0.0529025276	-2.3333572284
O	-1.4034640344	0.0368321998	2.2016343255
H	-2.2052984854	-0.4061595159	-0.0587502218
H	2.5005409099	0.2883572867	0.2639637273
H	1.4517958535	0.0772172476	-2.0424088279
H	1.0264151299	0.2903442019	2.1391262785

Table SI6 T_2/T_1 high energy structure

C	0.8002668159	0.1119140342	-1.1743167158
C	1.3788457698	-0.0336664781	0.0466649828
C	-0.6656485569	0.0883572830	-1.2864722391
C	-0.7151931031	-0.5963177508	1.0918772553
N	0.6295148397	-0.2805699601	1.1510402186
N	-1.3121262950	-0.1450909563	-0.0871059153
O	-1.2718065326	0.2912515326	-2.3071746123
O	-1.4129947211	-0.1093722458	2.2152865037
H	-2.3072638694	-0.2018047158	-0.1082885560
H	2.4375398624	0.0480140609	0.1952227719
H	1.3741838140	0.3098259514	-2.0556094585
H	1.0415980196	-0.3499502484	2.0534570394

Table SI7 S_1/S_0 structure

C	0.9138314022	0.1150844535	-1.3286052802
C	1.2463043998	0.0389527913	-0.0256774748
C	-0.4138445010	-0.0006409806	-1.9461486103
C	-0.8120103809	-0.0903399423	1.4443296299
N	0.5318453770	-0.1260099787	1.1512496459
N	-1.7053919177	-0.0369192189	0.4272308198
O	-0.6978339805	0.1315392614	-3.0725312413
O	-1.1778942484	-0.1225183826	2.6169653799
H	-2.6449128096	0.0376882257	0.7766098606
H	2.2931046865	0.1314378466	0.1943127816
H	1.7254521800	0.2641379807	-2.0191777352
H	1.0879212628	-0.1501933110	1.9755205595

Table SI8 S_1/S_0 structure

C	0.8404301371	-0.3882741751	-1.1389583670
C	1.3636572079	0.1611004925	0.0674264300
C	-0.5857994543	0.0948100138	-1.3190242999
C	-0.7473386644	-0.0411555896	1.1542623565
N	0.6605127255	0.0592852046	1.1905056201
N	-1.2910494841	0.0848755284	-0.0814214111
O	-1.1715423921	0.3494271373	-2.3221758923
O	-1.3647160093	-0.1215926294	2.1837695603
H	-2.2782138991	0.2316263999	-0.1058874162
H	2.2910735706	0.7052045013	0.1513107696
H	0.6730259586	-1.4579055247	-0.9703241588
H	1.0302088881	0.3084040275	2.0868172354

Table SI9 S_1/T_2 short N5-C4

C	0.7457583659E+000	0.8585690727E-001	-0.1183201812E+001
C	0.1346147156E+001	-0.4074750700E-001	0.2396776777E-001
C	-0.5167960921E+000	0.6932772144E-001	-0.1118376633E+001
C	-0.6437595464E+000	-0.7972080809E-001	0.1123490135E+001
N	0.5692747914E+000	-0.1316096344E-002	0.1262505280E+001
N	-0.1355777616E+001	0.1741267101E+000	-0.6314248402E-001
O	-0.1311447786E+001	-0.1571939522E+000	-0.2401015729E+001
O	-0.1370756094E+001	-0.3533765240E-001	0.2246496907E+001
H	-0.2272384323E+001	0.5820478720E+000	0.1321804244E+000
H	0.2486936668E+001	0.3932215300E-002	0.3908274574E-001
H	0.1219485075E+001	0.7086511446E-001	-0.2088980475E+001
H	0.9655046142E+000	-0.4158287705E+000	0.2062697490E+001

Table SI10 S_1/T_2 O oop at C2

C	0.7207193347E+000	0.5404413031E+000	-0.1041995827E+001
C	0.1293051248E+001	0.2508558334E-001	0.3011261906E-001
C	-0.6154067987E+000	0.7889541755E-001	-0.1258695509E+001
C	-0.8282376889E+000	-0.3515004446E+000	0.1160613517E+001
N	0.5334624999E+000	-0.4092007963E+000	0.1158342133E+001
N	-0.1234866879E+001	-0.1491886299E+000	-0.1005830249E+000
O	-0.1203965908E+001	-0.1907759917E+000	-0.2217057422E+001
O	-0.1261467845E+001	0.4650474832E+000	0.2169631328E+001
H	-0.2228318601E+001	0.5933996271E-001	-0.1969247412E+000
H	0.2237709505E+001	-0.9433396611E-001	-0.8868514851E-001
H	0.1326453843E+001	0.8814070647E+000	-0.1824347781E+001
H	0.7861837495E+000	-0.9232554540E+000	0.1959877674E+001

Table SI11 S_1/T_2 O oop at C4

C	0.7396965049E+000	-0.1274161535E+000	-0.1183581471E+001
C	0.1388072994E+001	0.1795664224E+000	0.2269321770E-001
C	-0.5781992765E+000	-0.4543799128E+000	-0.1318400552E+001
C	-0.7216274806E+000	0.5507630010E-001	0.1212343038E+001
N	0.6848163994E+000	-0.8784009430E-001	0.1230636108E+001
N	-0.1247175439E+001	-0.1222163572E+000	-0.9023621213E-001
O	-0.1305904312E+001	0.2902328226E+000	-0.2215681008E+001
O	-0.1489435326E+001	0.1172246811E+000	0.2227369294E+001
H	-0.2401489767E+001	-0.5029292654E-001	0.3884043548E-001
H	0.2379979558E+001	0.2074132672E+000	-0.1082453823E+000
H	0.1192788838E+001	0.6294225603E-001	-0.2229694600E+001
H	0.1140511933E+001	0.3651356730E+000	0.1969392159E+001

Table SI12 S_1/T_2 long C4=O

C	0.7955283799E+000	-0.4712930308E-003	-0.1138186338E+001
C	0.1396922546E+001	-0.1974190918E+000	0.1253005689E+000
C	-0.5096665194E+000	-0.8516847529E-001	-0.1125893902E+001
C	-0.7520747648E+000	-0.2432311834E-001	0.1230346239E+001
N	0.6395584381E+000	0.2005221134E+000	0.1206052218E+001
N	-0.1301760435E+001	0.8813605582E-001	-0.9003793366E-001
O	-0.1446808571E+001	0.4136930038E-001	-0.2446553456E+001
O	-0.1360621564E+001	-0.6746796206E-001	0.2207129882E+001
H	-0.2216487541E+001	-0.3063749243E+000	-0.2429819002E+000
H	0.2315955286E+001	0.3734969408E+000	0.1063863861E+000
H	0.1294637427E+001	0.1084697352E+000	-0.2055563425E+001
H	0.1273570240E+001	-0.1124242168E+000	0.1916455028E+001

Table SI13 S_1/T_2 short C4=O

C	0.8409049074E+000	0.1171948950E+000	-0.1162336059E+001
C	0.1340590124E+001	-0.7303719375E-001	0.9869219276E-001
C	-0.6481521120E+000	-0.2050382878E-001	-0.1282711744E+001
C	-0.5895163597E+000	-0.3850027103E+000	0.1088391699E+001
N	0.7486797280E+000	0.1650572314E+000	0.1220085361E+001
N	-0.1273173057E+001	-0.1367081100E+000	-0.5124466190E-001
O	-0.1442694424E+001	0.1863930571E-001	-0.2159941749E+001
O	-0.1485395379E+001	0.2387941691E+000	0.2135027779E+001
H	-0.2338778881E+001	-0.3342733334E+000	-0.3411325088E+000
H	0.2403144487E+001	0.3426371977E-001	0.2764713530E+000
H	0.1273598757E+001	0.3166421956E+000	-0.2082854764E+001
H	0.1178771033E+001	-0.1931103633E+000	0.1897542075E+001

Table SI14 S_1/T_1 structure

C	0.9836355077E+000	0.1406243980E+000	-0.1143500245E+001
C	0.1449914676E+001	0.5707801023E-001	0.3651861790E-001
C	-0.5452849909E+000	-0.5772411560E-001	-0.1259581177E+001
C	-0.7375776282E+000	-0.1628189517E-001	0.1402671376E+001
N	0.6593788002E+000	-0.1557165883E+000	0.1263188216E+001
N	-0.1252041450E+001	-0.3583168788E-001	-0.2286584457E-001
O	-0.1592511021E+001	-0.4494825768E-001	-0.2597838863E+001
O	-0.1440508019E+001	0.9104664586E-001	0.2244650522E+001
H	-0.2249992108E+001	0.3027744712E+000	-0.1262690327E-002
H	0.2379347100E+001	0.1716134697E+000	0.3079064309E+000
H	0.1540047148E+001	0.2653976913E-001	-0.2024912483E+001
H	0.9929001141E+000	-0.4397883799E-001	0.2182080141E+001

Table SI15 S_2 minimum

C	0.7967212453	-0.0280301979	-1.2071382112
C	1.4517341779	0.1012369344	0.1217244735
C	-0.5724707897	-0.0221614792	-1.2368639933
C	-0.7504279119	-0.0959085385	1.1913794609
N	0.6012557846	0.0681464962	1.2101861859
N	-1.2872425539	-0.0766844651	-0.0860996226
O	-1.3922484407	-0.0084333215	-2.3037524707
O	-1.4375686809	-0.2084183329	2.1727637907
H	-2.2827409095	-0.0940349516	-0.1529556493
H	2.3269732293	0.7124067528	0.2606681141
H	1.3702542326	-0.0271992266	-2.1081978724
H	0.9830459640	0.1475766133	2.1278974254
